# Features Importance Analysis of Diesel Vehicles’ NO_x_ and CO_2_ Emission Predictions in Real Road Driving Based on Gradient Boosting Regression Model

**DOI:** 10.3390/ijerph182413044

**Published:** 2021-12-10

**Authors:** Hung-Ta Wen, Jau-Huai Lu, Deng-Siang Jhang

**Affiliations:** 1 Department of Mechanical Engineering, National Chung—Hsing University, Taichung City 402, Taiwan; jhlu@dragon.nchu.edu.tw; 2 Taichung Motor Vehicles Office, Taichung City 432, Taiwan; Shane0316@hotmail.com

**Keywords:** NO_x_ and CO_2_ emission, diesel vehicle, gradient boosting regression, features importance, PEMS

## Abstract

In order to have an accurate and fast prediction of the artificial intelligence (AI) model, the choice of input features is at least as important as the choice of model. The effect of input features selection on the emission models of light diesel vehicles driven on real roads was investigated in this paper. The gradient boosting regression (GBR) model was used to train and to predict the emissions of nitrogen oxide (NO_x_), carbon dioxide (CO_2_), and the fuel consumption of real driving diesel vehicles in urban scenarios, the suburbs, and on highways. A portable emissions measurement system (PEMS) system was used to collect data of vehicles as well as environmental conditions. The vehicle was run on two routes. The model was trained with the first route data and was used to predict the emissions of the second route. There were ten features related to the NO_x_ model and nine features associated with the CO_2_ model. The importance of each feature was sorted, and a different number of features were used as input to train the models. The best NO_x_ model had the coefficient of determination (R^2^) values of 0.99, 0.99, and 0.99 in each driving pattern (urban, suburbs, and highways). Predictions of the second route had the R^2^ values of 0.88, 0.89, and 0.96 respectively. The best CO_2_ model had the R^2^ values of 0.98, 0.99, and 0.99 in each driving pattern, respectively. Predictions of the second route had the R^2^ values are 0.79, 0.82, and 0.83, respectively. The most important features for the NO_x_ model are mass air flow rate (g/s), exhaust flow rate (m^3^/min), and CO_2_ (ppm), while the important features for the CO_2_ model are exhaust flow rate (m^3^/min) and mass air flow rate (g/s). It is noted that the regression models based on the top three features may give predictions very close to the measured data.

## 1. Introduction

The problem of air pollution in many metropolises has been gaining more and more attention recently. According to the report of the World Health Organization (WHO), about 7 million people died of diseases related to air pollution in 2018 [1]. Among the pollutants, nitrogen oxides (NO_x_) are severely concerning because they not only have a harmful effect on the human body but are also closely related to ozone (O_3_) formation in the troposphere and on the ground. Kuo et al. proposed research on the effects of ambient air pollutants on childhood asthma hospitalization. They found that the O_3_ was positively associated with this childhood disease [2]. Zhong et al. studied the effects of air pollutants on human health. The results also indicated that the O_3_ had the highest health impact, followed by particulate matter (PM_10_ and PM_2.5_) [3]. In 2021, Wang et al. proposed an investigation on air pollution during pregnancy and childhood autism spectrum disorder (ASD). The results indicated that the O_3_ had significant positive associations with childhood ASD and might have different effects before and after birth [4].

There are many anthropogenic sources of NO_x_. The combustion of diesel engines is one of the primary sources of NO_x_ in many areas [5]. The abatement of NO_x_ in the transportation sector has become an important issue.

In order to reduce the emission of nitrogen oxides effectively, both the automobile manufacturer and governments are betting on considerable research and development expenses, and the regulations of vehicle emissions are becoming more stringent. For example, the EURO 6 emission standard implemented by the European Union in 2014 is a milestone for the regulation of diesel vehicles [6]. It indicates that the government and industry must face the air pollution problem seriously.

It is required that all new vehicles should comply with the current emission standard. As the regulatory standards are becoming stricter and stricter, new vehicles will become cleaner and cleaner. Taking EURO 6 as an example, the emission standard of NO_x_ is only 0.08 g/km. However, studies of the emissions measured by the portable emission measurement system (PEMS) show that the actual emissions in real road driving are much higher than the emission standards [7]. The year 2000 model diesel vehicles met the regulation standards of 0.5 g/km NO_x_ in the laboratory test. However, the study found that the actual emissions of diesel vehicles on real roads reached 1.0 g/km. For the year 2014 model diesel vehicles, the legal limit was reduced to 0.08 g/km in the laboratory test, about 1/6 of the standard in 2000. However, the actual road emission in NO_x_ was about 0.6 g/km. The regulation was tightened by 85% in 14 years, but the real road emissions did not decrease as much as expected. They were reduced by only 40%. Research conducted by Vicente et al. collected the real road emission data of a total of 541 EURO 5 and EURO 6 vehicles in Britain, Germany, France, and the Netherlands. The results of this investigation showed that only 10% of the EURO 6 vehicles might meet the EURO 6 standard, and 13% of vehicles exceeded by ten times the EURO 6 standards [8].

Therefore, the emissions of diesel vehicles in real road driving becomes an important issue, and the PEMS measurement becomes a valuable method for emission regulation. The impact of the vehicle emission control policy can be effectively evaluated by this method since the driving pattern on a real road is quite different from that in the laboratory. The US EPA also recommends PEMS as an accepted alternative to the laboratory-based chassis dynamometer measurement methods [9]. However, the real road measurement is time-consuming and the cost is very high, so it is helpful to develop a new method to predict the emissions of diesel vehicles on real roads to reduce the measurement cost [10]. Thus, using artificial intelligence techniques is a prospective method to cope with these problems.

Using PEMS measurements for emission evaluation has gained much attention recently, and many investigations have been conducted. Frey et al. proposed protocols of data collection, screening, processing, and analysis to assure data quality and to provide insights regarding the quantification of hot-stabilized emissions [11]. Frey et al. used PEMS to investigate the impact of routes, time of day, and road grade on the emissions of selected light duty gasoline vehicles [12].

McCaffery et al. conducted a study using PEMS to measure 50 heavy-duty vehicles and analyzed these data in the Emission Factor (EMFAC) model [13]. The investigation found that the temperature of selective catalytic reduction (SCR) influenced NO_x_ emissions strongly, and exhaust temperature showed the opposite trend.

The main objective of this paper is to find a feasible way to build a predictive model for diesel emissions. The emissions in a running vehicle are affected by many factors, including the engine operating conditions, the vehicle characteristics, and the environmental conditions. It will be a huge computational burden if all parameters are taken into account. Sorting of the relative importance for all the parameters is conducted in this research. It was found that three parameters are good enough to build a model. A lot of computational costs could be saved with this simplified model. However, the ranking order of the input features is not the same for different routes. The results of this paper would be very useful for the modelers, thereafter.

**Figure 1:**
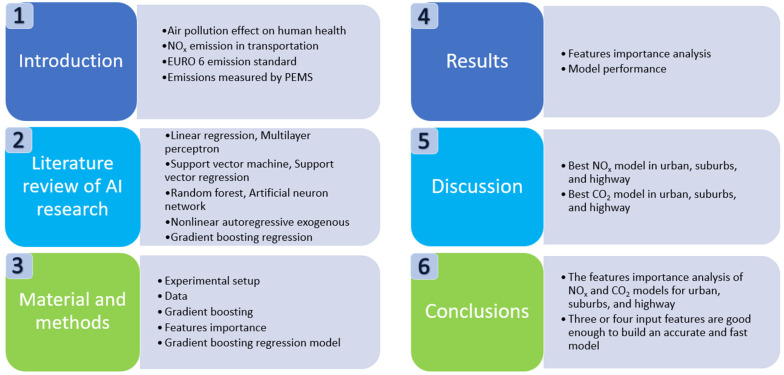
Structure of this study.

A structure diagram, Figure 1, of this article is presented to make it easier to read this paper.

## 2. Literature Review of AI Research

Jaworski et al. presented an analysis of emission data from the PEMS system for real driving cycles of various types of vehicles, complying with EURO 2–EURO 6 standards and created the emission models with the Regression Learner applications in MATLAB R2018b. Among the methods of regression trees and support vector technologies that have been used, the boosted regression tree model was assessed to be the most appropriate to well represent the real data [14].

Donateo et al. proposed a neural network model based on the interpolation of the time-histories of driving conditions (speed, altitude, ambient temperature, humidity, and pressure) and emissions measured on a diesel start-and-stop vehicle while performing a series of real driving emission (RDE) tests. The Bayesian optimizer, implemented in the MATLAB environment, was used to deal with the high number of combinations. This method minimized the number of combinations examined by the model and significantly reduced the computational time [15].

As mentioned above, a lot of PEMS measurements in real road driving have been conducted before, but not many of them have used artificial intelligence (AI) to analyze the collected data and to establish models for further predictions and evaluations. The purpose of this study is to investigate the technique of AI models for diesel vehicles in real road driving to reduce the cost of calculation and to increase the accuracy of the model prediction.

The big data analysis technique is an important tool to reduce the cost of emission measurements on real roads. Compared with traditional ways of data analysis, the analysis of vast amounts of data is no longer difficult in today’s big data era. For regression analysis, machine learning technology has been widely used in related topics, such as support vector machine (SVM), random forest (RF), and artificial neural networks (ANN).

Zeng et al. used SVM to estimate the fuel consumption of gasoline vehicles and found a relationship between the fuel consumption and the corresponding factors such as average speed and driving distance [16]. They also used the multiple linear regression (MLR) model and artificial neural network to link the fuel summation with the SVM model for comparisons. However, the measurement period took one month, which is too much of a cost in time.

Henrik Almer compared the effect of sampling rate on the model performance. He used the vehicle data, road data, and weather data collected in one year between 1 June 2013 and 31 May 2014 for training. The data collected between 1 June 2014 and 31 October 2014 were used for validation and testing. The results of comparison showed that the sampling rate of 10 min is better than the sampling rate of 1 min in model performance because of its smaller variance in predicting fuel consumption. Moreover, among machine learning models (linear regression, random forest, support vector regression (SVR), and ANN), the performance of the random forest model was the best [17].

L. Thibault et al. presented the use of information and communication technology (ICT) to transmit driving information to the cloud system through smartphones and to calculate pollutant emissions in real time [18]. This coupled a microscopic model with a real-world speed profile to estimate on-road pollutant emissions. However, it is not possible to use the existing microscopic model well for a large vehicle fleet because the input parameters of this model are not available for all vehicles. Thus, the modeling approach should be chosen according to the vehicle data available for each car.

Air pollution is one of the major concerns for citizens due to its impact on public health. Vehicle emissions are related to vehicle technology and driver behavior. Therefore, L. Thibault et al. created a vehicle IoT device that gives the driver real-time feedback about the emissions and reminds the driver of exposure during the trips [19]. The whole system includes an IoT device, a smartphone, and web-based simulation models. It provides a chance to improve the existing emissions through driving behavior.

Alimissis et al. used two interpolation methodologies to evaluate the ANN model and MLR model of urban air quality data. The predictions of air pollutants’ concentrations were compared with statistical measurements. ANN models were found to have better performance than MLR models in most cases [20]. G. Bandyopadhyay and S. Chattopadhyay also compared the performance of the ANN model and MLR model for ozone data. They found that ANN models are perfect for an interpolation solution to nonlinear problems using one input variable [21].

M. Gardner and S. Dorling used multilayer perceptron (MLP) neural networks to model the NO_x_ pollutants and found that they perform well in predicting hourly variations of NO_x_ and NO_2_ [22]. This indicates that MLP neural networks are capable of coping with complex patterns of source emissions.

In addition, F. Perrotta et al. proposed a big data-type analysis of the driving fuel consumption records of 1010 trucks over a 300 km travel distance, and performed a large amount of data processing using three techniques (SVM, RF, ANN) in a machine learning model [23]. They successfully established a fuel consumption model with big data and found that the RF model outperformed the other two models.

The gradient boosting regression model is a very powerful tool in modeling and prediction, and has been applied in many different fields [24,25,26,27,28]. However, this model has not been used in pollution predictions widely. A review conducted in 2018 by Bai et al. [29] showed that many AI methods have been used for air pollution predictions, including ANN, adaptive neuro-fuzzy (ANF), MLP, SVM, and SVR. However, the GBR model was not included. An investigation of the predictive power of the GBR model was carried out in this paper. Wen et al. proposed another ANN nonlinear autoregressive exogenous model (NARX) to predict NO_x_ emissions in real road driving [30]. It is an inexpensive way of conducting RD emission measurements using an NGK NO_x_ sensor and an Arduino board with Can Bus to measure the instantaneous vehicle emissions on the road. The results of measurement seemed to be quite reasonable compared with a full PEMS system.

Yun et al. proposed a real-time model to predict vehicle instantaneous emissions such as NO_x_ and CO_2_ [31]. This is a practical model that integrates an ANN model with a vehicle dynamic model. The accuracy of both NO_x_ and CO_2_ models were evaluated by varying number of features.

Table 1 summarizes the key points of previous investigations conducted by other researchers related to the AI model applications in vehicle emissions measurements.

The process of this study is to establish the AI model of one vehicle based on the PEMS data of that specific vehicle in urban, suburb, and highways situations and then use the data to predict the emission and fuel consumption for the second route of the same vehicle. One vehicle was used in this study. The model performance of the vehicle includes NO_x_ and CO_2_ emissions and fuel consumption.

The method of gradient boosting regression (GRB) was adopted in this study to model the NO_x_ and CO_2_ emissions in three routes (urban, suburbs, and highway) and use them to predict the NO_x_ and CO_2_ emissions and fuel consumption of the same vehicle on a second journey. A different number of input features were chosen for comparison to determine the best combinations of input features of the models.

## 3. Materials and Methods

### 3.1. Experimental Setup

The HORIBA OBS-ONE PEMS was used in this research for emission measurements on the real road. It is vehicle-mounted pollution analysis equipment that can be used to measure CO, CO_2_, THC, NO_x_, and PN in driving vehicles continuously. The system also integrates with the satellite positioning system (Global Positioning System, GPS) and the environmental conditions such as atmospheric temperature, humidity, atmospheric pressure, etc., to obtain the actual emissions data when the vehicle is driving on real roads. The specifications of the PEMS are listed in Table 2 [32]. There are 35 parameters that can be collected during the real road testing.

**Figure 2:**
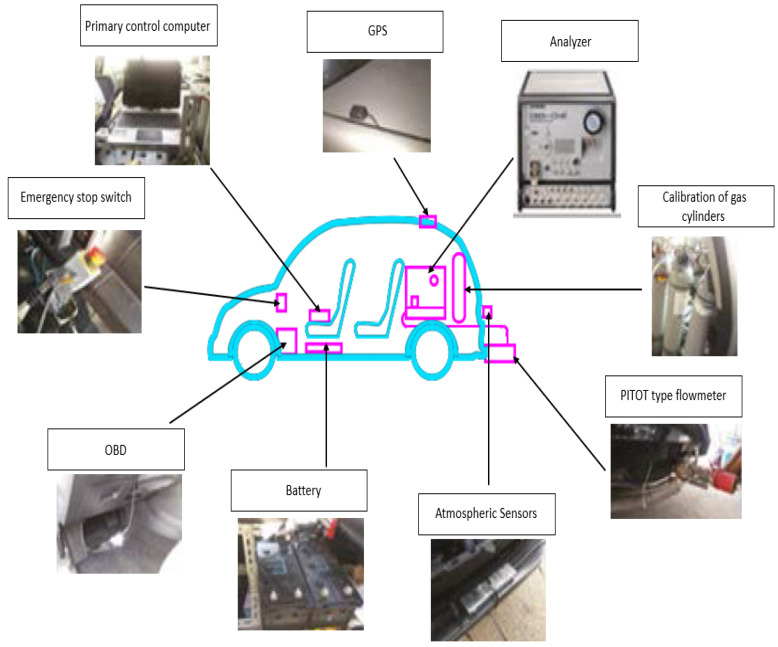
Detailed settings of PEMS installation [10].

The detailed settings of PEMS installation are shown in Figure 2, including the location of the analyzer, pitot tube flowmeter, atmospheric sensors, tailpipe temperature sensor, and primary control computer [10]. Figure 2 shows the installation of the system in the cabin of the test vehicle. The function of the primary control computer is using the HORIBA default software to collect the data from different sensors, store the data, and output the raw data.

The PEMS system was powered by an independent battery pack inside the cabin. All the instruments were well grounded to the negative pole of the battery to prevent any pulse damage. Moreover, standard gases were carried in the cabin for calibration of sensors before and after each test to ensure the accuracy of measurement.

The raw data were processed in the following way. Take the NO_x_ data as an example. The direct measurements by the PEMS system include the instantaneous concentrations of NO_x_ in the exhaust flow ($x_{NO_{x}}$), the exhaust temperature ($T_{exh.}$), the pressure in the exhaust pipe ($P_{exh.}$), and the exhaust flow rate ($Q_{ex}$). The density of NO_x_ ($\rho_{NO_{x}}$) was obtained using the equation of state for ideal gas with the exhaust temperature and the exhaust pressure. The instantaneous concentrations of NO_x_ can then be converted to the mass flow rate by multiplying the exhaust mass flow rate as shown by Equation (1).

(1) $${\dot{m}}_{NO_{x}}=Q_{ex}x_{NO_{x}}\rho_{NO_{x}}$$

**Figure 3:**
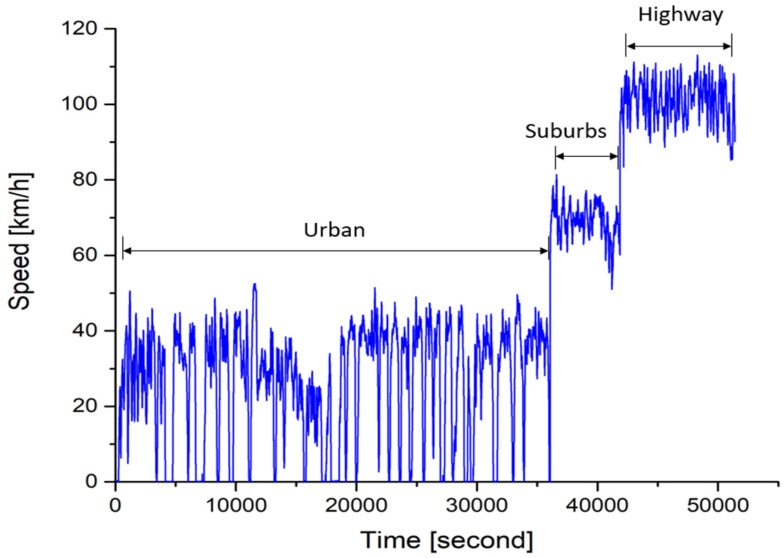
Speed variations of testing vehicle in route 1 of the real road.

One light duty diesel vehicle, Peugeot 208, produced in 2015, which meets EURO 5 emission standards, was used as the testing vehicle in this study. The engine displacement of this vehicle is 1.6 L. The testing vehicle was run on real roads with two routes. The driving route started from Zhangbin Industrial Park to Lukang, Puyan, and Huawei. The total distance of this route is about 64 km. The route was divided into three parts intentionally, including urban, suburbs and, highways. The vehicle speed in each part was well controlled in specific ranges. The driving speed was from 0 to 60 km/h in the urban, from 60 to 90 km/h in suburbs, and above 90 km/h on highways. Figure 3 shows the speed variations of route 1. It is noted that vehicle speed varies a lot in the first part because of lots of traffic lights in the urban area. In the second part, we can see that the vehicle speed was much faster than that in the urban area. No traffic lights were observed in this part. In the last part of this route, the vehicle speed was in the range of 90~110 km/h. This is the highway part. However, the speed was not fixed in a constant value because acceleration and deceleration occurred quite often during overtaking, and the average speed was above 100 km/h.

Two vehicle routes were used in this study. The driving patterns of these two routes are shown in Table 3. It is noted that these two routes are not different by very much. The distance and the average speed were controlled so as not to deviate from each other. The driving data on the urban, suburbs, and highways sections were analyzed in this paper. One used for building the model, and the other was used for validating the model.

### 3.2. Data

The total time span for one route was about 90 min. The sampling rate of data collection was 10 H_z_. The 10 H_z_ sampling rate is fast enough to capture the transient behavior of the vehicle in acceleration or deceleration. A higher sampling rate is feasible for the hardware capability of the PEMS system. However, it will cause a big burden for the following data analysis because a total of 35 data were collected at a time. In total, about 54,000 data sets could be collected in one testing. There were, in total, 35 raw data collected in the PEMS. The raw data can be divided into three groups. One is operating parameters of the vehicle, including exhaust volume flow rate (m^3^/min), exhaust temperature (°C), exhaust pressure (kPa), engine coolant temperature (°C), fuel pressure (kPa), engine speed (rpm), intake air temperature (°C), mass air flow rate (g/s), fuel rail pressure (kPa), fuel rail pressure (direct inject) (kPa), commanded EGR (%), barometric pressure (kPa), fuel rail pressure (absolute) (kPa), engine oil temperature (°C), engine fuel rate (L/h), actual engine percent torque (%), and engine reference torque (Nm). The second group is the environmental parameters of the vehicle, including test time (s), test distance (km), GPS altitude (m), GPS latitude (deg), GPS longitude (deg), GPS speed (km/h), GPS course (deg), system battery voltage (V), ambient temperature (°C), ambient relative humidity (%), and ambient pressure (kPa). The third group is the outcome of the vehicle, including CO (ppm), CO_2_ (ppm), H_2_O (%), NO (ppm), NO_x_ (ppm), NO_2_ (ppm), and THC (ppmC).

Since the goal of this research is to build the models of CO_2_ and NO_x_, some parameters that are not closely related to the formation of CO_2_ and NO_x_ are not considered in this study to reduce the burden of model building.

**Figure 4:**
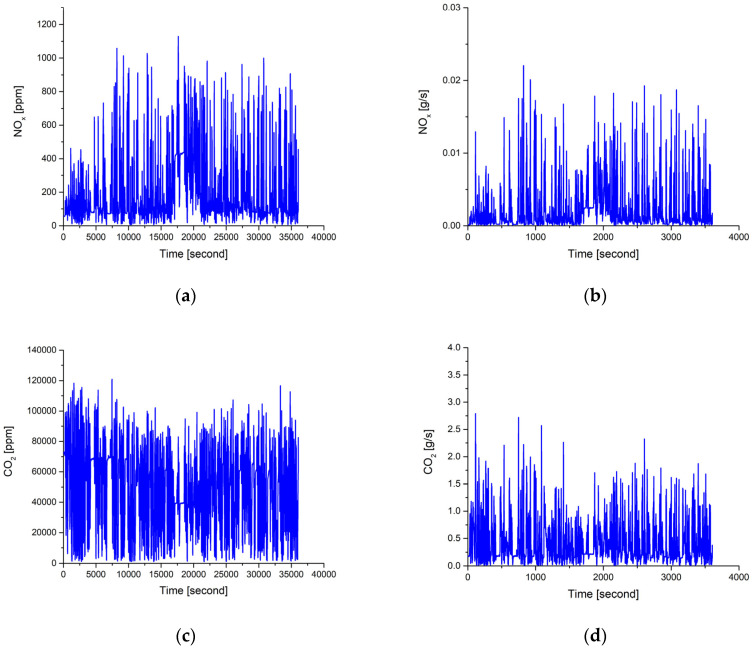
The instantaneous concentrations and the production rates of NO_x_ and CO_2_ in urban part. (**a**) NO_x_ concentrations (ppm); (**b**) NO_x_ production rates (g/s); (**c**) CO_2_ concentrations (ppm); (**d**) CO_2_ production rates (g/s).

The instantaneous NO_x_ and CO_2_ concentrations in the tailpipe are shown in Figure 4a,c. The raw data were recorded with a sampling rate of 10 Hz. A total of about 36,070 data are presented in this Figure. It is noted that the NO_x_ concentrations in the urban part vary a lot at different vehicle speeds. The same trend could be found in suburbs and highways. Even on the highway, the vehicle speeds are quite stable, and the NO_x_ concentrations vary a lot too. The variations of CO_2_ concentrations are similar to NO_x_ emissions mentioned above. The maximum values of CO_2_ are about 120,000 ppm (12%), which corresponds to an air–fuel ratio of about 20 at full loads. The minimum values of CO_2_ are about 1093 ppm, which corresponds to very lean combustion at idle condition. It is noted that the CO_2_ concentrations of diesel engines are much lower than of gasoline counterparts because diesel engines run at much leaner conditions [33].

The instantaneous concentrations can be converted to the mass flow rate by multiplying the exhaust mass flow rate by Equation (1). The results of the conversion calculation are shown in Figure 4b,d. Because of the limited space, only the variations in urban area are shown. It is noted that the maximum flow rate of NO_x_ is about 0.022 g/s, which occurs at acceleration. The minimum flow rate of NO_x_ is about 0.00004 g/s, which occurs at deceleration. For CO_2_, the maximum flow rate is about 2.79 g/s, which occurs at acceleration, and the minimum flow rate is about 0.0068 g/s, which occurs at deceleration.

The instantaneous flow rate can be integrated with time to obtain the total emission production factor. The results of the integration are shown in Table 4.

### 3.3. Gradient Boosting

An introduction of gradient boosting was presented by Li [34]. This indicated that the gradient boosting consists of two sub-terms, gradient descent and boosting, and it could be applied in regression, classification, and ranking. These applications are based on the gradient boosting algorithm proposed by Friedman [35]. This algorithm is used to minimize the loss function of the model by adding weak learners using gradient descent. The regression function is shown in Equation (2). The ensemble of gradient boosting is a supervised learning algorithm that can learn nonlinear functions to solve regression and classification problems. It is a complex model built by combining several simple models in the best possible way. The high bias and low variance models can be combined additively to form an ensemble with reducing bias while maintaining the low variance in boosting.

(2) $$f_{m}\left(x\right)=f_{m-1}\left(x\right)+\rho_{m}h\left(x;\alpha_{m}\right)$$

where f(x) is the loss function and h(x;α) is the base-learner model. For each iteration *m* = 1, 2, ⋯, M, compensating the residues is equivalent to optimizing the expansion coefficients $\rho_{m}$ and $\alpha_{m}$.

### 3.4. Features Importance

There are 35 parameters that were collected by the PEMS during the RD testing. Nine of them are closely related to the formation of NO_x_, and they are considered as the input features for the NO_x_ model, including mass air flow rate (g/s), exhaust flow rate (m^3^/min), CO_2_ (ppm), engine speed (rpm), tailpipe exhaust temp (°C), GPS speed (km/h), ambient humidity (%), ambient temperature (°C), and GPS altitude (m). As for the CO_2_ model, eight input features were considered for building the model, including exhaust flow rate, mass air flow rate, engine speed, GPS speed, tailpipe exhaust temperature, ambient humidity, GPS altitude, and ambient temperature.

Since there are too many input features for the NO_x_ model as well as the CO_2_ model, a way of reducing input features should be considered to reduce the calculation burden. John et al. proposed the two main categories of reducing selected feature numbers [36]. One is the wrapper method that pluses and/or reduces the features to figure out the optimizer that could obtain the highest performance. The other is the filter method that evaluates the correlation between the variable and the predicted value, and excludes the least relevant variable.

Casimir et al. used the Sequential Backward Selection (SBS) algorithm to find the most relevant features [37]. The appropriate features are determined for better accuracy. Dewi et al. proposed a method to cope with the features selection by using random forest (RF) algorithm [38]. They found out that different combinations of features created different model accuracy. As a result, the model performance is higher after using the RF features selection algorithm. The model performance statistic results of using four datasets (Wisconsin Cancer, Forest Fire, Wine Quality, and Bike Sharing) show that more input features do not guarantee obtaining higher performance because more features add more complexity to the model.

There are ten features (nine features from PEMS and acceleration derived from GPS speed) that were selected for the NO_x_ model and nine features (eight features from PEMS and acceleration derived from GPS speed) for the CO_2_ model. The rank and score of feature importance were generated by permutation techniques. This was written in python code by importing the permutation_importance from sklearn library directly. The results showed their relative predictive power to the model. The process is used to break the input feature values by randomly shuffling. This step might decrease the model performance. If the decrease is small, it means the performance of the model is pretty good. Furthermore, if the decrease is large, there is a large impact on predictions.

### 3.5. Gradient Boosting Regression Model

Python is an interactive, object-oriented programming language. It has hundreds of excellent libraries for developers to use. Python offers a programming language that is stable, flexible, and has tools available. Therefore, there are lots of Python AI applications today. The GBR model was built in Python code. The coding process includes preprocessing, data-loading, model-defining, and model-fitting.

In this study, the data are divided into two subsets, the training set and the testing set for building the GBR model. The percentage of each data set is listed in Table 5. In general, the train–test split procedure is used to estimate the performance of the model. In this study, the training data are 75%, and the testing data are 25%.

In building the GRB model, several parameters should be assigned in the beginning. The main parameters for the NO_x_ model are listed in Table 6. The parameters of the GBR model include the following: N_estimators denote the number of trees used for boosting, and the default value is 100. Its value was set at 500 in this model. More estimators may obtain better model performance but require more calculation cost. Max_depth denotes the maximum depth of the tree, and the default value is 3. It was set at 12 in this code. Min_samples_split denotes the minimum number of samples needed to split an internal node, and it was set to 10. Max_features denote the number of features to consider when finding the best split, and the number is the square root of total features. The subsample denotes the fraction of samples that can be used to fit the individual base learners, and the default value is 1.0. Its value was set at 0.8 in this model. Learning_rate denotes a learning rate that determines the step size at each iteration while moving toward a minimum of a loss function and the value was set at 0.1. Loss denotes the loss function. Ls was selected in this code and meant the least square loss function. There is another loss function that can be selected such as lad (least absolute deviation), huber (combination of ls and lad).

**Figure 5:**
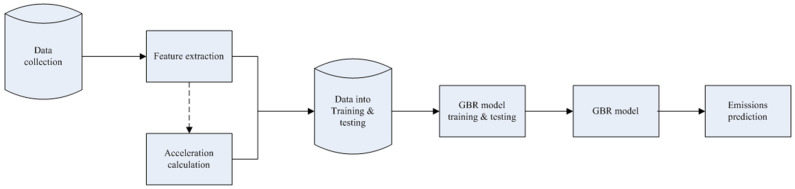
Flowchart of proposed method for emissions prediction.

During the training process of the GBR model, the ensemble was imported from Python module sklearn (SciKit-Learn) and using the gradient boosting regressor defined with an ensemble. The GBR model was defined with the above parameters and was used to fit the experimental data. Figure 5 shows the flowchart of the proposed method for emissions prediction. The first step is collecting data from PEMS records. Then, feature extraction is conducted that uses the features selection method to determine the appropriate features for model input. It is noted that acceleration is not the raw data collected by PEMS directly. It needs to be calculated from the GPS speed. The third step is loading the data and splitting them into training and testing sub-datasets by fraction. Then starts the process of training and testing for the model. The fifth step is finishing the compile process and obtaining the model. Finally, the model is used to predict the emissions in the second route.

The model performance is examined by using mean absolute error (MAE), root means square error (RMSE), and the coefficient of determination $R^{2}$. The statistical equations are given in Equations (3)–(5), respectively, where P_i_ is the predicted value obtained with the model and T_i_ is the measured value from PEMS. ${\bar{P}}_{i}$ is the average of the predicted value for the whole dataset.

(3) $$\mathrm{MAE}=\frac{1}{n}\;\sum_{i=1}^{n}\left|T_{i}-P_{i}\right|$$

(4) $$\mathrm{RMSE}=\sqrt{\frac{1}{n}\;\sum_{i=1}^{n}{\left(T_{i}-P_{i}\right)}^{2}}$$

(5) $$R^{2}=1-\frac{\;\sum_{i=1}^{n}{\left(P_{i}-T_{i}\right)}^{2}}{\sum_{i=1}^{n}{\left({\bar{P}}_{i}-T_{i}\right)}^{2}}$$

## 4. Results

### 4.1. Features Importance Analysis

**Figure 6:**
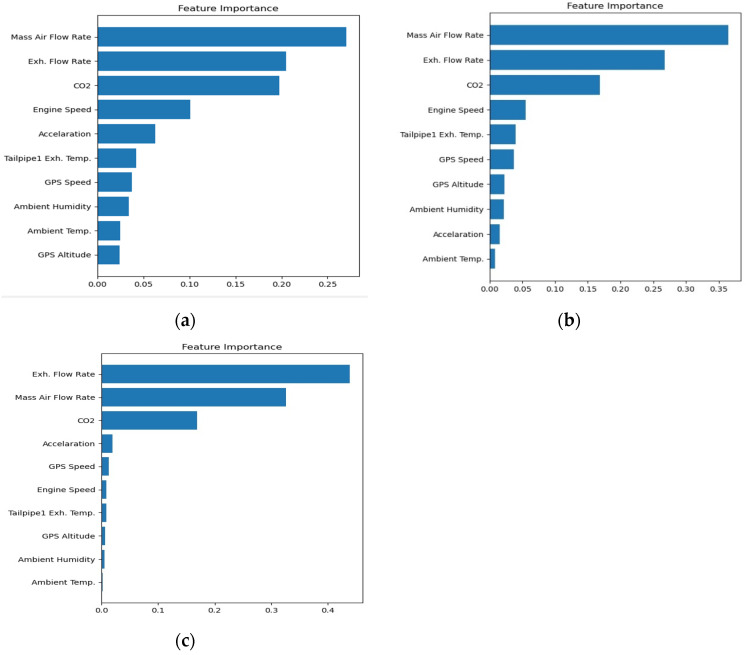
Feature importance detailed scores of NO_x_ model (**a**) Urban; (**b**) Suburbs; (**c**) Highway.

The detailed scores ranking of feature importance for NO_x_ models in the urban, suburb, and highway driving patterns are shown in Figure 6. It is noted that the top three features of NO_x_ models are the same for urban, suburban, and highways. They are mass air flow rate, exhaust flow rate, and the concentration of CO_2_. It is not surprising that mass air flow rate is the most important feature because it is closely related to engine load, and NO_x_ formation is known to be dominantly determined by engine load. The second important feature is exhaust flow rate, which is actually the sum of mass air flow rate and mass fuel flow rate. Air–fuel ratio is also known to be an important factor to determine the formation of NO_x_. No wonder it is the second important feature. The third important feature is the concentration of CO_2_ in the exhaust flow. CO_2_ is one of the products of combustion, just as NO_x_. However, unlike the gasoline engine in which CO_2_ concentration in the exhaust flow is almost a constant value, the concentration of CO_2_ in diesel engines reflects the air–fuel ratio as well as the engine load. As a result, the CO_2_ concentration is also a good feature for the NO_x_ model.

The fourth important feature in the urban, suburb and highway patterns is not the same for all. It is interesting to note that engine speed is important in urban and suburbs driving, while acceleration is important on the highways. Engine speed is related to vehicle speed. We can see that vehicle speed varies a lot in both urban and suburbs driving, as shown in Figure 6. However, the vehicle speed is almost fixed in a narrow range on highways. That is the reason engine speed is not so important on highways.

All of the other features are not important relative to the first four features. The order of importance of the last six features in urban, suburb, and highways patterns are not the same either. Since the importance of the last six features is much lower than the first four, the order of these features is actually not to be discussed in this study.

Table 7 shows the ranking of all features in urban, suburbs, and highways driving again for a clear comparison. It is noted that even though the actual order of the last six features is not exactly the same, the trend of descending of the order is quite similar. It could be concluded that the relative importance of the features of the NO_x_ model in urban, suburban, and highways driving are quite close to each other.

**Figure 7:**
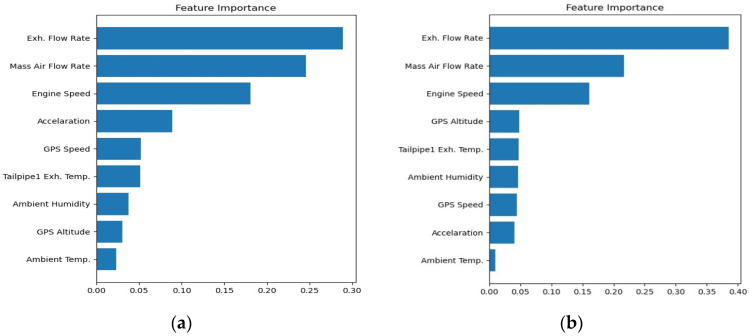

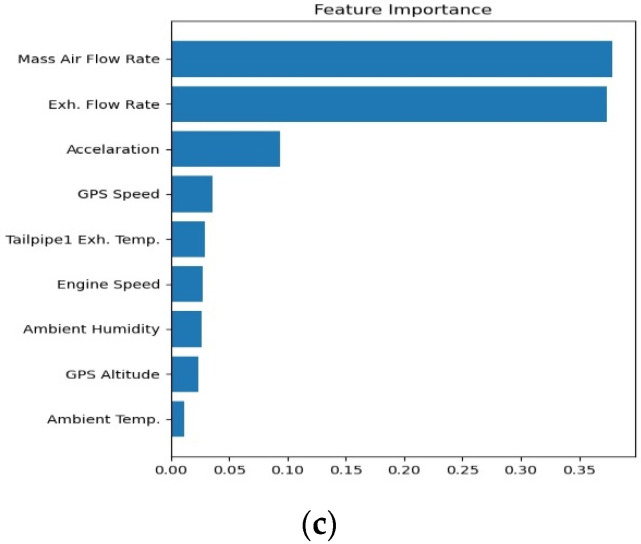
Feature importance detailed scores of CO_2_ model (**a**) Urban; (**b**) Suburbs; (**c**) Highway.

The number of features is nine for the CO_2_ model. All of the features are the same as the NO_x_ model, except that CO_2_ is excluded. The ranking of feature importance of CO_2_ models for urban, suburbs, and highways patterns are shown in Figure 7. It is noted that the top two features of CO_2_ models are the same for urban, suburban, and highways driving. They are mass air flow rate and exhaust flow rate. This is reasonable because these two features are closely related to engine load and air–fuel ratio, and the CO_2_ concentration is determined by air–fuel ratio, while the CO_2_ production rate is determined by engine load.

The third important features in urban, suburbs and highways are not the same. It is interesting to note that engine speed is important in urban and suburbs driving, while acceleration is important in highways, just as was the case in the NO_x_ model. The reason is also the same. Vehicle speed varies a lot in both urban and suburbs driving, but it is almost fixed in a narrow range in highways driving. That is the reason engine speed is not so important on highways.

Nine input features were considered in the CO_2_ model. However, not every feature is important for prediction. According to our calculation, the importance of each feature is ranked in Table 8. It is noted that the order of relative importance is not the same in the urban, suburbs, and highways driving patterns. Table 8 shows the ranking of all features in urban, suburbs, and highways again for a clear comparison. The first three features contribute the most (above 70%) importance from Figure 7. This also means the other six features contribute to a lesser extent. It is noted that even though the actual order of the last six features is not exactly the same, the trend of descending of the order is quite similar. It could be concluded that the relative importance of the features of the CO_2_ model in urban, suburbs, and highways driving are quite close to each other.

### 4.2. Model Performance

In this study, ten input features and nine input features were selected for NO_x_ and CO_2_ models, respectively, and the targets were NO_x_ emission gram per second (g/s) and CO_2_ emission gram per second (g/s). The statistics of model performance for NO_x_ and CO_2_ in the three parts of route 1 are listed in Table 9 and Table 10. The R^2^ value is an important index to determine how well the model fits the raw data. The feature importance rank shown in Table 7 and Table 8 is used to check the fitting of the model. It is noted that the feature importance rank of urban driving was selected as the base, and the same order was used in suburbs and highways. It is found that for the NO_x_ model, the R^2^ values of train data are all very close to 1.0 in all three parts of route 1. As for the test data, the R^2^ values of the urban part increase from 0.78 to 0.98 as the number of features increase from 3 to 10. The more input features we use, the higher value of R^2^ we have. It is a reasonable result since more features are used to model the raw data, it is expected that more characteristics of data can be captured. However, it is worthwhile to note that there is a big gap between three features and four features, and the R^2^ values vary very little as feature numbers increase more. This implies that four features are the minimum number to totally grasp the characteristics of NO_x_ in the urban area. In the suburbs part and the highways part, the same trends of R^2^ values occur. The gap between three features and four features is not so big as that in urban and in highways, but it is still obvious that four features are the minimum number to totally grasp the characteristics of NO_x_ in the suburbs area and in the highways area.

The performance of CO_2_ models is similar to that of NO_x_ models. The R^2^ values of train data are all very close to 1.0 in all three parts of route 1, implying that the training process goes very well. As for the test data, the R^2^ values of the urban part increase from 0.80 to 0.95 as the number of features increase from three to nine. However, unlike the NO_x_ model where a big gap between three features and four features can be observed, the R^2^ values increase almost linearly with the number of features up to seven features where the increase in the R^2^ value begins to slow down. In the suburbs part and the highways part, the same trends of R^2^ values occur. The R^2^ values increase linearly with the number of features up to six or seven features. It seems that the CO_2_ model needs more input features to totally grasp the characteristics of raw data in all parts of the route.

**Figure 8:**
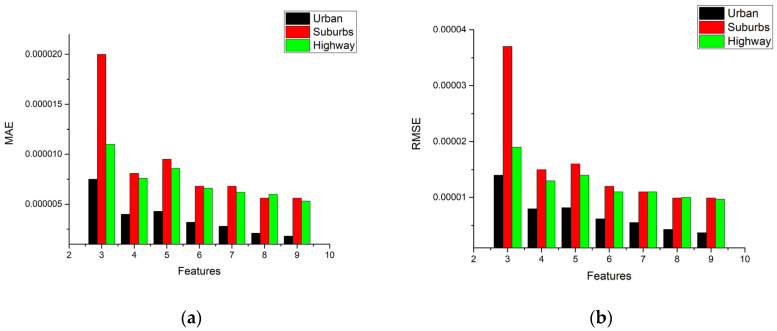
Performance MAE and RMSE statistics results of NO_x_ model (**a**) MAE; (**b**) RMSE.

In addition, Figure 8a,b show the performance statistics results of MAE and RMSE for NO_x_ models in route 1, respectively. They are other methods of performance evaluation used to check the model accuracy. An opposite trend of the R^2^ value is found, that the more features we use, the lower value of MAE we obtain. A lower value of MAE means better accuracy by the definition of MAE in Equation (3). It is noted that the prediction values are very close to the target values. In the meantime, the results for RMSE statistics are very similar to those of MAE.

In Figure 8a, it is noted that the value of MAE decreases as the number of features increases in urban, suburbs, and highways routes. Four features might be the best choice for this case considering the computational cost and accuracy, but for reaching the best performance, all features are the only option, even when using more calculation cost. It is noted that the trend in Figure 8b is the same as MAE.

**Figure 9:**
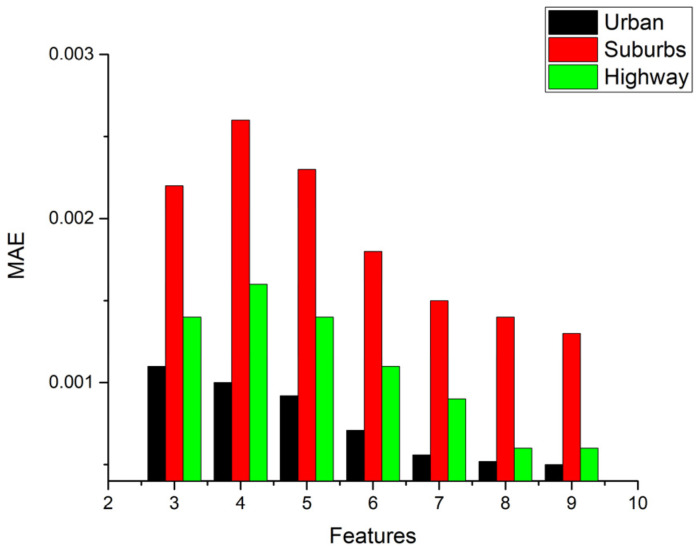
Performance MAE statistics results of CO_2_ model.

Figure 9 shows the MAE statistics for the CO_2_ model. Only the MAE statistics are shown because the trend of RMSE statistics is also very similar to that of MAE, just as the case of the NO_x_ model. It is interesting to note that the trend of MAE in the urban part is the same as that in the NO_x_ model, i.e., the value of MAE decreases as the number of features increases. However, in the suburbs and highways parts, different trends show that three features input has a lower value of MAE than four features input. As a result, using three features would be the best choice to obtain good performance and low computation cost for urban, suburbs, and highways driving in route 1. Increasing the number of features can obtain better performance if computational cost needs are not to be considered. In that case, eight features might be the best choice.

In this study, the NO_x_ and CO_2_ models obtained in route 1 were used to predict the vehicle emissions driving in the urban, suburbs, and highways parts of the second route to investigate the universality of the models. The results of NO_x_ predictions in the second route are listed in Table 11. It is noted that the R^2^ values in the urban part vary from 0.69 to 0.88. The R^2^ values of route 2 are lower than route 1, even for the same vehicle. This is reasonable because the traffic flow, route conditions, and driver behaviors are different. The average speed of these two routes is quite similar, as shown in Table 3, but the acceleration of these two routes are different. The second route has wider acceleration ranges than route 1, probably reflecting the characteristics of the drivers. Taking a close look at the model performance, the lowest R^2^ value occurs when using the top three features for input features. This is the same trend as for the route 1 urban NO_x_ model. Unlike route 1’s urban performance results, the maximum R^2^ values were found using six and seven features for input, and the whole results show that the performance does not increase while the number of features increases.

In the suburbs part of the second route, the range of R^2^ varies from 0.79 to 0.89 and shows a different trend to the urban part. The maximum R^2^ value uses five features for input, and then the R^2^ values drop after five features and reach the lowest value when using all features for input. The R^2^ of the highways part varies from 0.93 to 0.96. All of these values are higher than the urban and suburbs values. It is noted that the R^2^ of highways is the best among the three driving patterns.

The performance results when using route 1 CO_2_ models to predict the second route CO_2_ are listed in Table 12. The performance value increases while using three and four input features and reaches the maximum value of using five features, then drops with an increasing number of features for urban driving. The trend of R^2^ variations agrees with the results of the CO_2_ models feature importance analysis shown in Figure 7a. It is noted that the features importance influences the model performance a lot and reflects the same trend of using this model to predict the second route.

According to the above results, GBR models present excellent R^2^ values, representing the model performance for NO_x_ and CO_2_ prediction.

Table 12 shows that the R^2^ values of suburbs driving are higher than for urban. This is the same result as using the route 1 NO_x_ model to conduct prediction in the second route due to the similar feature importance, such as mass air flow rate, exhaust flow rate, and engine speed. It is interesting that the trend of R^2^ values of highway driving using three, four and five features are similar to urban, but the R^2^ values of highway driving using six, seven and eight features are similar to suburbs.

**Figure 10:**
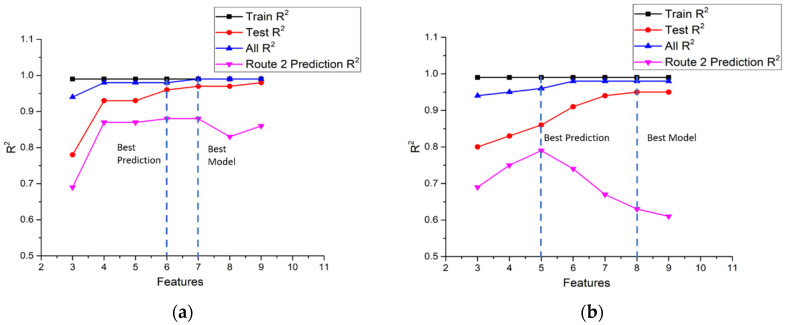
(**a**) Comparisons of the R^2^ value of NO_x_ models for the training sub-dataset, the test sub-dataset, all data, and the route 2 prediction in urban; (**b**) Comparisons of the R^2^ value of CO_2_ models for the training sub-dataset, the test sub-dataset, all data, and the route 2 prediction in urban.

**Figure 11:**
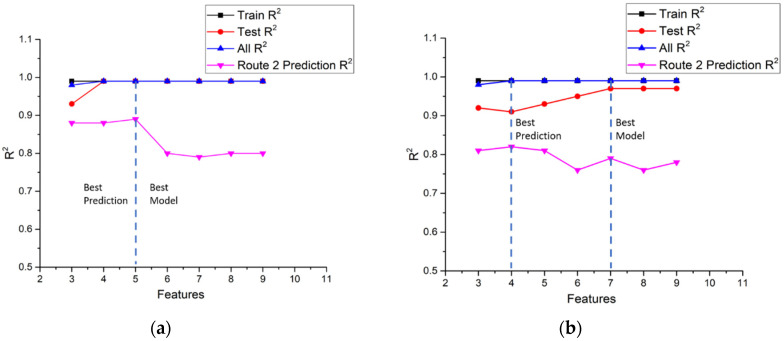
(**a**) Comparisons of the R^2^ value of NO_x_ models for the training sub-dataset, the test sub-dataset, all data, and the route 2 prediction in suburbs; (**b**) Comparisons of the R^2^ value of CO_2_ models for the training sub-dataset, the test sub-dataset, all data, and the route 2 prediction in suburbs.

**Figure 12:**
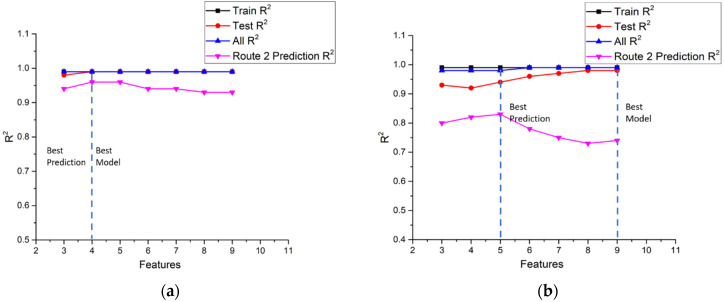
(**a**) Comparisons of the R^2^ value of NO_x_ models for the training sub-dataset, the test sub-dataset, all data, and the route 2 prediction in highway; (**b**) Comparisons of the R^2^ value of CO_2_ models for the training sub-dataset, the test sub-dataset, all data, and the route 2 prediction in highway.

In summary, Figure 10, Figure 11 and Figure 12 shows the comparisons of the R^2^ values of NO_x_ and CO_2_ models for the training sub-dataset, the test sub-dataset, all data, and the prediction of the second route using a different number of features in urban, suburbs, and highways according to Table 9, Table 10, Table 11 and Table 12. The criterion for the best model is to obtain the highest R^2^ value in both route 1 and the second route. The best prediction is the highest R^2^ value of the second route. For example, the best NO_x_ model in urban driving, as shown in Figure 10a, is the model with seven input features. However, the best prediction is the model with six input features. Figure 10b shows the best model with eight input features and the best prediction with five input features for the CO_2_ model in urban driving, according to the same criterion. The statistics of the input number of features for the best model and best prediction in urban, suburbs, and highways routes are listed in Table 13. It can be found that the number of input features for the best prediction is less than that of the best model.

**Figure 13:**
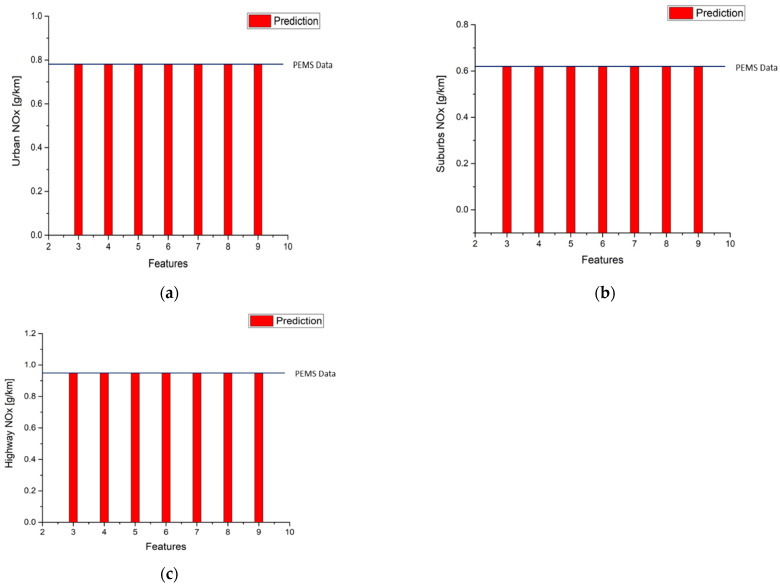
Results of NO_x_ emission factor predictions (**a**) Urban; (**b**) Suburbs; (**c**) Highway.

The models trained by GBR can not only predict the instantaneous emission production on the road, as shown in previous figures, but also can be used to evaluate the emission factors of the vehicle driven on real roads. Table 14 and Figure 13 present the results of the calculation for the NO_x_ emission factors with different input features. It is noted that the predicted emission factors are very close to the measured factors in the three parts of route 1 for all the cases of 3 input features to 10 input features.

**Figure 14:**
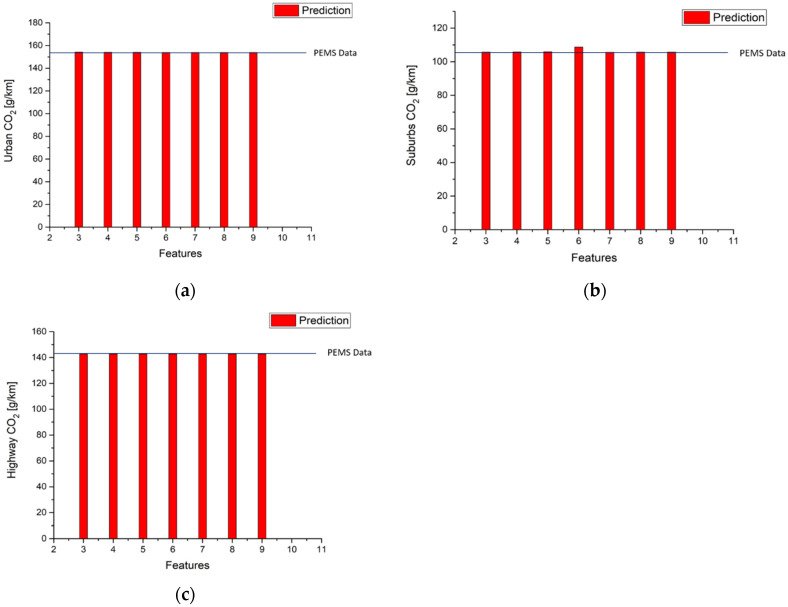
Results of CO_2_ emission factor predictions (**a**) Urban; (**b**) Suburbs; (**c**) Highway.

Table 14 and Figure 14 show the results of CO_2_ emission factors calculations. The results are as good as those for NO_x_ predictions except in the suburbs part with six input features where the error between the predicted value and measured data is 0.4%, which is still very small.

**Figure 15:**
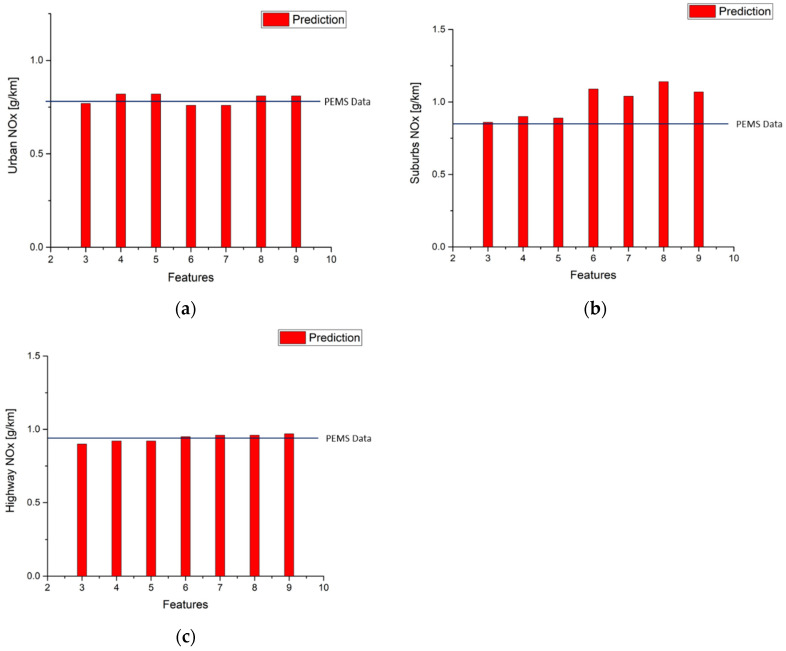
Second route NO_x_ emission factor predictions by using route 1 NO_x_ urban model (**a**) Urban; (**b**) Suburbs; (**c**) Highway.

**Figure 16:**
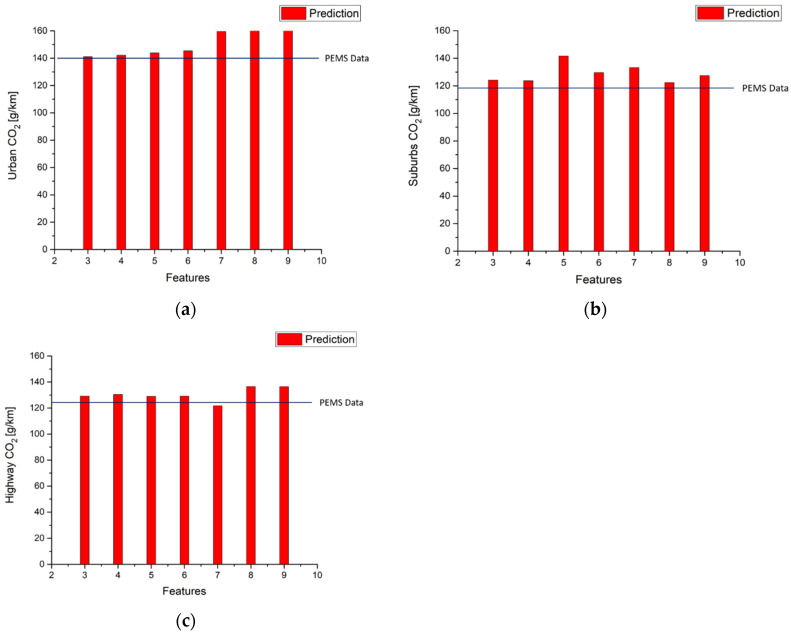
Second route CO_2_ emission factor predictions by using route 1 using CO_2_ urban model (**a**) Urban; (**b**) Suburbs; (**c**) Highway.

Table 15, and Figure 15 and Figure 16 show that using route 1 NO_x_ and CO_2_ models to predict urban, suburbs, and highway emissions in the second route obtains the same results as Table 11 and Table 12 in terms of emissions gram per kilometer. It is clear that model performance decides the error between measurement data and predictions. The best performances of NO_x_ and CO_2_ in the second route urban model use the top six features and top five features, respectively. The best performance of NO_x_ prediction in the second route in the suburbs uses the top five features but CO_2_ prediction uses the top four features. The best performance of the NO_x_ prediction in the second route in the highway model uses the top four features, and using the top five features to fit the model obtains the best performance of CO_2_ prediction in the highway model.

Diesel is a volatile substance that consists of hydrocarbon. The diesel fuel chemical formula is $C_{16}H_{30}$ with the density of 0.85 kg/L. The stoichiometric combustion reaction of diesel fuel with air in a diesel engine is as following.

(6) $$C_{16}H_{30}+23.5\left(O_{2}+3.76N_{2}\right)\to 16CO_{2}+15H_{2}O+88.36N_{2}$$

**Figure 17:**
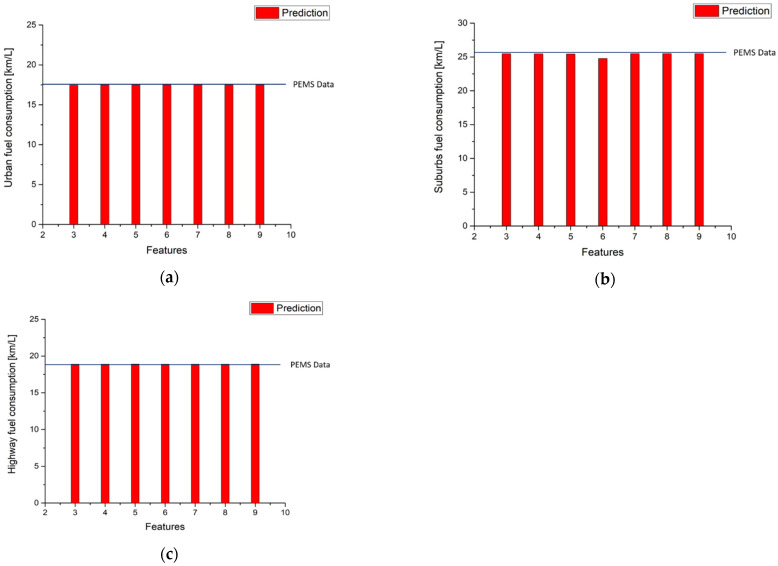
Fuel consumption statistics results in route 1 (**a**) Urban; (**b**) Suburbs; (**c**) Highway.

**Figure 18:**
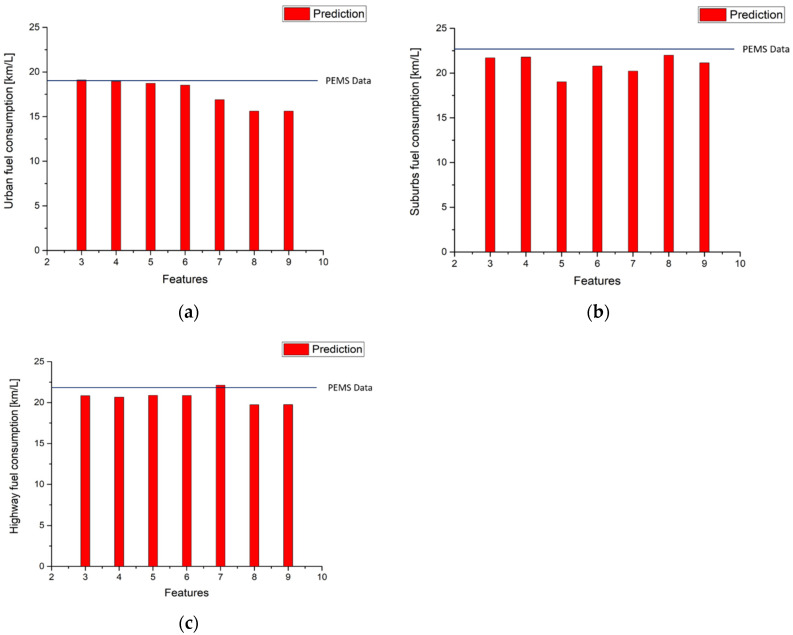
Second route fuel consumption predictions by using route 1 CO_2_ urban model (**a**) Urban; (**b**) Suburbs; (**c**) Highway.

According to Equation (6), fuel consumption can be calculated by utilizing CO_2_ emission gram per kilometer, as obtained above. The results of fuel consumption calculations in route 1 are shown in Table 16 and Figure 17. It is noted that the errors of fuel consumption are very similar to the errors of CO_2,_ as in Figure 12. This is very reasonable because the fuel consumption in a diesel engine is closely related to CO_2_ production. The results of fuel consumption predictions in the second route are shown in Table 16 and Figure 18. The same trends of model errors can be observed as in Figure 16.

## 5. Discussion

**Figure 19:**
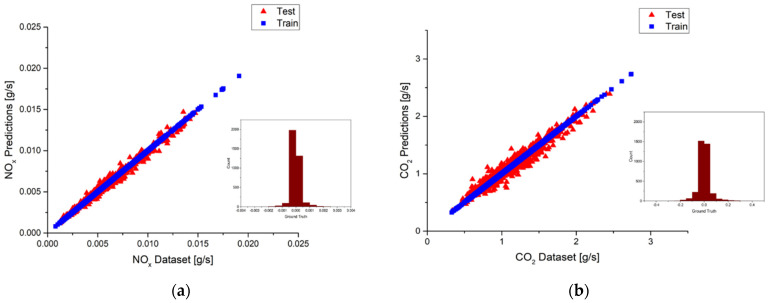
(**a**) Deviations between the predictions and the measurements and ground truth plot of the best NO_x_ model in urban; (**b**) Deviations between the predictions and the measurements and ground truth plot of the best CO_2_ model in urban.

According to Table 9 and Table 10, the best model is built by selecting the top seven input features for the NO_x_ model and the top eight input features for the CO_2_ model in urban driving. The R^2^ values of the NO_x_ model and the CO_2_ model are 0.99 and 0.98, respectively. These results are the best R^2^ values among the NO_x_ and CO_2_ models when selecting different input numbers of features. It is noted that the choice was based on the prediction accuracy only, calculation cost was not considered. The prediction results of the best NO_x_ and CO_2_ models are plotted in Figure 19a,b, in which the red triangle represents the test sub-dataset and the blue square represents the training sub-dataset. Since the R^2^ value of the test sub-dataset for the best NO_x_ model is 0.97, some small deviations between the predictions and the measurements can still be observed in Figure 19a. Furthermore, the bar chart located at the lower right corner shows the ground truth of the predictions. Ground truth is an evaluation of the results of model accuracy against the targets. The distribution of ground truth would concentrate on zero value for a perfect model. It can be seen for the best NO_x_ model the distribution of ground truth spreads a little bit around zero value, indicating the existence of deviations. Figure 19b shows the predictions of the best CO_2_ model. The same deviations can be observed as those in the NO_x_ model. However, since the R^2^ value of the test sub-dataset for the best CO_2_ model is 0.95, more deviations can be observed in Figure 19b. The bar chart of the ground truth also spreads more in the CO_2_ model. In general, both the NO_x_ and CO_2_ models show pretty good accuracy, as shown in Figure 19a,b.

**Figure 20:**
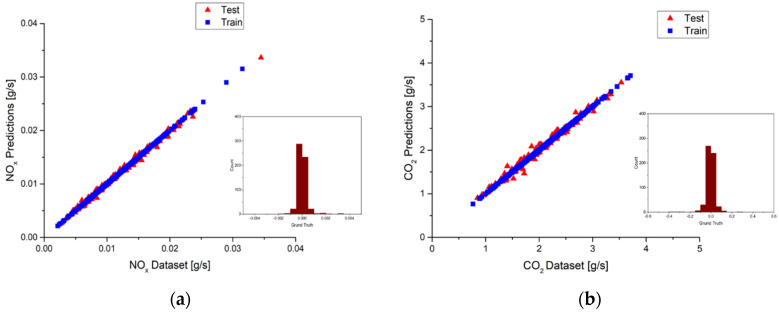
(**a**) Deviations between the predictions and the measurements and ground truth plot of the best NO_x_ model in suburbs; (**b**) Deviations between the predictions and the measurements and ground truth plot of the best CO_2_ model in suburbs.

As for the suburbs part, the best models are built by using the top five features for NO_x_ and the top seven features for CO_2_ according to the results listed in Table 9 and Table 10, respectively. The R^2^ values of both the NO_x_ model and the CO_2_ model are 0.99. Figure 20a,b shows the prediction results of the best NO_x_ and CO_2_ models. The red triangle represents the test sub-dataset, and the blue square represents the training sub-dataset. It can be observed that deviations are very small in the NO_x_ models because the R^2^ value is close to 1. However, since the R^2^ value of the test sub-dataset for the best CO_2_ model is 0.97, more deviations can be observed in Figure 20b. Taking a close look at the ground truth distributions, they are very close to the zero value, indicating only little deviations occur. Both the NO_x_ and CO_2_ models show pretty good accuracy, as shown in Figure 20a,b.

**Figure 21:**
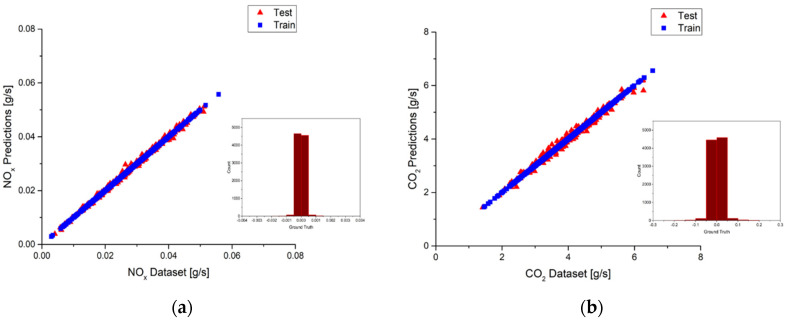
(**a**) Deviations between the predictions and the measurements and ground truth plot of the best NO_x_ model in highways; (**b**) Deviations between the predictions and the measurements and ground truth plot of the best CO_2_ model in highways.

The model performance in the highways part is examined in the following. The best model is built by using the top four features for NO_x,_ and the top nine features are used for the CO_2_ model according to the results listed in Table 9 and Table 10, respectively. It is noted that the R^2^ values of both the NO_x_ model and the CO_2_ model are 0.99. The best NO_x_ and CO_2_ models prediction results of the test and training sub-datasets are plotted in Figure 21a,b with a red triangle and blue square, respectively. Very few deviations can be observed for the best NO_x_ model in Figure 21a. More deviations can be found for the best CO_2_ model in Figure 21b because the R^2^ value is 0.98. The bar charts located in the lower right corner show the ground truth distributions of the predictions. Both models show pretty good accuracy, with the distributions concentrating around zero value.

**Figure 22:**
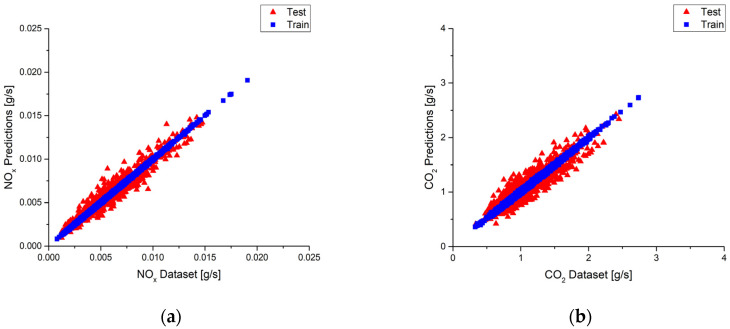
(**a**) Deviations between the predictions and the measurements plot of using the top 4 features NO_x_ model in urban. (**b**) Deviations between the predictions and the measurements plot of using the top 4 features CO_2_ model in urban.

Figure 22a shows the deviations between the predictions and the measurements by using the top four features as input to build the NO_x_ model in the urban part. Comparisons between Figure 19a and Figure 22a show that the deviations in Figure 22a are a little bit greater than Figure 19a. This is reasonable since the best model has the highest performance R^2^ value. It is also noted that a similar result as the NO_x_ model is found for the CO_2_ model in the urban part shown in Figure 22b. Moreover, taking a look at the second route prediction R^2^ values for the NO_x_ model in the urban route would find that the R^2^ values of using the top four features for input are close to the R^2^ value of the best prediction. This is similar to the CO_2_ model where the R^2^ values of using the top four features for input is 0.75, and the best prediction has the R^2^ value of 0.79. As a result, selections of the NO_x_ model and CO_2_ model that use the top four features could be the second choice for second route prediction.

The purpose of this paper is to find a feasible way to build a predictive model for diesel emissions. It was found that three parameters are good enough to build a model. A lot of computational costs could be saved with the simplified model. The results of this paper would be very useful for the modelers thereafter. The strategy of emission reduction is not the purpose of this paper. However, according to the results of the emission model developed in this paper, the most important features are exhaust flow rate, air–fuel ratio, and engine speed. The implications of the findings are that reducing the engine load may abate the emissions of a running vehicle.

The NO_x_ emission factors calculation from Table 14 shows that the lowest value occurs in the suburbs. It is observed from Table 14 that there is the same trend in the CO_2_ emission factors calculation. The common reason might be acceleration and deceleration. It would be helpful to remind drivers to control their speed to reduce air pollution.

## 6. Conclusions

The features importance analysis of NO_x_ and CO_2_ models for urban, suburb, and highways are built successfully. The top three features of urban, suburban, and highway NO_x_ models are airflow rate, exhaust flow rate, and CO_2_ concentrations, and the top three features of urban and suburban CO_2_ models are exhaust flow rate, airflow rate, and engine speed. The top three features of highway CO_2_ models are airflow rate, exhaust flow rate, and acceleration.

In order to have an accurate prediction, generally, we need more input features. However, the accuracy is not proportional to the number of input features. More input features do not guarantee more accurate predictions. The best models need 4~9 input features to have the highest R^2^ value. The best predictions of the second route need 4~6 input features. The choice of input features depends on the route characteristics and the emissions.

If the computational cost is a major concern, the model could be simplified to reduce the number of input features. The R^2^ values of the simplified NO_x_ models using the top three and top four input features are very close to the best model. The difference in R^2^ values is as small as 0.01. The R^2^ values of the simplified CO_2_ models using the top three and top four input features are also very close to the best model. The difference in R^2^ values is about 0.04.

The purpose of this paper is to find a feasible way to build a predictive model for diesel emissions. It was found that three parameters are good enough to build a model. A lot of computational costs could be saved with the simplified model. The results of this paper would be very useful for the modelers thereafter.

Moreover, the gradient boosting regression model is a very powerful tool in modeling and prediction, and has been applied in many different fields. However, this model has not been used in pollution predictions widely. The NO_x_ and CO_2_ GBR models were built successfully in this study to predict the emissions of diesel vehicles on real roads with pretty good R^2^ values. The results show that the GBR is a practical approach to making accurate predictions.

It is recommended that three or four input features are good enough to build an accurate and fast model for the prediction of the NO_x_ and CO_2_ emissions of diesel vehicles running on real roads. However, the choice of input features is important. An inappropriate choice of input feature may give poor results.

## Figures and Tables

**Table 1:** Key point of each study in the literature review.

No.	Authors	Reference	Key Point
1	Donateo et al.	[15]	Proposed a neural network model based on the interpolation of the time-histories of driving conditions.
2	Zeng et al.	[16]	Used support vector machine (SVM), multiple linear regression, and artificial neural network (ANN) models.
3	Henrik Almer	[17]	Proposed machine learning models (linear regression, random forest, SVR, and ANN) and found that the performance of random forest model was the best.
4	Alimissis et al.	[20]	Found artificial neural networks had better performance than multiple linear regression in most cases.
5	Bandyopadhyay et al.	[21]	Used artificial neural network (ANN) models as an interpolation solution to nonlinear problems.
6	Gardner et al.	[22]	Indicated that MLP neural networks were capable of coping with complex patterns of source emissions.
7	Perrotta et al.	[23]	Performed a large amount of data processing using three techniques (SVM, RF, ANN) in the machine learning model.
8	Wen et al.	[30]	Proposed ANN nonlinear autoregressive exogenous model (NARX) model to predict NOx emissions.
9	Yun et al.	[31]	Proposed a real-time model that integrates an artificial neural network (ANN) model with a vehicle dynamics model.

**Table 2:** Specifications of HORIBA OBS-ONE PEMS [32].

Species	Measurement Range	Measurement Principle
CO_2_	0–5~20 vol%	HNDIR
NO_x_	0–100~3000 ppm	HCLD
Rpm capture: OBD/ECU; Exh. volume: pitot tube meter; weight: 32 kg Dimension: 350(W) × 25(H) × 470(D) (mm); Vehicle type: Gasoline/diesel vehicles Power: 24DC/110AC; Regulations: CFR Part 1065 subpart J/EC NO 582/2011/UN ECE R83		

It is noted that the PEMS has the capability to measure five pollutants in exhaust pipe. However, only CO_2_ and NOx are used in the analysis of this study, so only the specifications of CO_2_ and NOx are listed in Table 2.

**Table 3:** Driving pattern of the testing vehicle on real roads.

Driving Pattern	Driving Distance		Acceleration		Average Speed	
	Route 1	Route 2	Route 1	Route 2	Route 1	Route 2
Urban	26.64 km	26.53 km	−6.7~5.3 m/s^2^	−7.0~7.4 m/s^2^	26.59 km/h	25.17 km/h
Suburbs	11.08 km	11.04 km	−3.9~5.5 m/s^2^	−4.8~3.6 m/s^2^	69.02 km/h	67.36 km/h
Highways	26.53 km	26.79 km	−7.3~7.7 m/s^2^	−7.5~6.5 m/s^2^	101.43 km/h	100.97 km/h

**Table 4:** Total emission rate.

Emissions	Urban	Suburbs	Highway	Average
NO_x_	0.78 g/km	0.62 g/km	0.95 g/km	0.78 g/km
CO_2_	154.12 g/km	105.54 g/km	142.68 g/km	134.11 g/km

**Table 5:** Training and test sets.

Driving Pattern	NO_x_		CO_2_	
	%	Instances	%	Instances
urban Training Test	75 25	27,052 9018	75 25	27,052 9018
suburbs Training Test	75 25	4337 1446	75 25	4337 1446
highways Training Test	75 25	7059 2353	75 25	7059 2353

**Table 6:** GBR model parameters.

N_Estimators: 500
Max_depth: 12
Min_samples_split: 10
Max_features: sqrt
Subsample: 0.8
Learning_rate: 0.1 Loss: ls

**Table 7:** Feature importance rank of NO_x_ model in urban, suburbs, and highway.

Feature	Rank		
	Urban	Suburbs	Highway
Mass Air Flow Rate	1	1	2
Exhaust Flow Rate	2	2	1
CO_2_	3	3	3
Engine Speed	4	4	6
Acceleration	5	9	4
Tailpipe Exhaust Temp	6	5	7
GPS Speed	7	6	5
Ambient Humidity	8	8	9
Ambient Temp	9	10	10
GPS Altitude	10	7	8

**Table 8:** Feature importance rank of urban, suburbs, and highway CO_2_ model.

Feature	Rank		
	Urban	Suburbs	Highway
Exh. Flow Rate	1	1	2
Mass Air Flow Rate	2	2	1
Engine Speed	3	3	6
Acceleration	4	8	3
GPS Speed	5	7	4
Tailpipe Exh. Temp	6	5	5
Ambient Humidity	7	6	7
GPS Altitude	8	4	8
Ambient Temp	9	9	9

**Table 9:** Performance R^2^ statistics results of NO_x_ model.

Selected Features	Urban			Suburbs			Highway		
	R^2^			R^2^			R^2^		
	Train	Test	All	Train	Test	All	Train	Test	All
Top 3 features	0.99	0.78	0.94	0.99	0.93	0.98	0.99	0.98	0.99
Top 4 features	0.99	0.93	0.98	0.99	0.98	0.99	0.99	0.99	0.99
Top 5 features	0.99	0.93	0.98	0.99	0.98	0.99	0.99	0.99	0.99
Top 6 features	0.99	0.96	0.98	0.99	0.99	0.99	0.99	0.99	0.99
Top 7 features	0.99	0.97	0.99	0.99	0.99	0.99	0.99	0.99	0.99
Top 8 features	0.99	0.97	0.99	0.99	0.99	0.99	0.99	0.99	0.99
Top 9 features	0.99	0.98	0.99	0.99	0.99	0.99	0.99	0.99	0.99

**Table 10:** Performance R statistics results of CO_2_ model.

Selected Features	Urban			Suburbs			Highway		
	R^2^			R^2^			R^2^		
	Train	Test	All	Train	Test	All	Train	Test	All
Top 3 features	0.99	0.80	0.94	0.99	0.92	0.98	0.99	0.93	0.98
Top 4 features	0.99	0.83	0.95	0.99	0.91	0.98	0.99	0.92	0.98
Top 5 features	0.99	0.86	0.96	0.99	0.93	0.98	0.99	0.94	0.98
Top 6 features	0.99	0.91	0.98	0.99	0.95	0.99	0.99	0.96	0.99
Top 7 features	0.99	0.94	0.98	0.99	0.97	0.99	0.99	0.97	0.99
Top 8 features	0.99	0.95	0.98	0.99	0.97	0.99	0.99	0.98	0.99
Top 9 features	0.99	0.95	0.98	0.99	0.97	0.99	0.99	0.98	0.99

**Table 11:** Performance statistics results of using NO_x_ models to predict the second route NO_x_ emission.

Selected Features	R^2^	R^2^	R^2^
	Urban Model	Suburbs Model	Highway Model
Top 3 features	0.69	0.88	0.94
Top 4 features	0.87	0.88	0.96
Top 5 features	0.87	0.89	0.96
Top 6 features	0.88	0.80	0.94
Top 7 features	0.88	0.79	0.94
Top 8 features	0.83	0.80	0.93
Top 9 features	0.86	0.80	0.93

**Table 12:** Performance statistics results of using CO_2_ models to predict the second route CO_2_ emission.

Selected Features	R^2^	R^2^	R^2^
	Urban Model	Suburbs Model	Highway Model
Top 3 features	0.69	0.81	0.80
Top 4 features	0.75	0.82	0.82
Top 5 features	0.79	0.81	0.83
Top 6 features	0.74	0.76	0.78
Top 7 features	0.67	0.79	0.75
Top 8 features	0.63	0.76	0.73
Top 9 features	0.61	0.78	0.74

**Table 13:** Input number of features for best model and best prediction.

	NO_x_			CO_2_		
	Urban	Suburbs	Highways	Urban	Suburbs	Highways
Best model	7	5	4	8	7	9
Best prediction	6	5	4	5	4	5

**Table 14:** Results of NO_x_ and CO_2_ emission factor predictions.

Selected Features	Urban		Suburbs		Highway	
	NO_x_ Prediction	CO_2_ Prediction	NO_x_ Prediction	CO_2_ Prediction	NO_x_ Prediction	CO_2_ Prediction
Top 3 features	0.78	154.24	0.62	105.77	0.95	142.76
Top 4 features	0.78	154.05	0.62	105.83	0.95	142.72
Top 5 features	0.78	154.01	0.62	105.94	0.95	142.63
Top 6 features	0.78	153.91	0.62	108.79	0.95	142.76
Top 7 features	0.78	153.95	0.62	105.70	0.95	142.77
Top 8 features	0.78	153.97	0.62	105.72	0.95	142.70
Top 9 features	0.78	153.97	0.62	105.72	0.95	142.60
PEMS data	0.78	154.12	0.62	105.54	0.95	142.60

**Table 15:** Second Route NO_x_ and CO_2_ emission g/km predictions statistics results of using route 1 urbanmodels.

Selected Features	Urban		Suburbs		Highway	
	NO_x_ Prediction	CO_2_ Prediction	NO_x_ Prediction	CO_2_ Prediction	NO_x_ Prediction	CO_2_ Prediction
Top 3 features	0.77	141.11	0.86	124.24	0.90	129.28
Top 4 features	0.82	142.21	0.90	123.73	0.92	130.39
Top 5 features	0.82	143.9	0.89	141.75	0.92	129.04
Top 6 features	0.76	145.44	1.09	129.67	0.95	129.20
Top 7 features	0.76	159.57	1.04	133.34	0.96	121.81
Top 8 features	0.81	172.54	1.14	122.44	0.96	136.55
Top 9 features	0.81	172.58	1.07	127.40	0.97	136.47
PEMS data	0.78	141.2	0.85	119.14	0.94	124.20

**Table 16:** Fuel consumption km/L statistics results of route 1 and route 2.

Selected Features	Urban		Suburbs		Highway	
	Route 1 Prediction	Route 2 Prediction	Route 1 Prediction	Route 2 Prediction	Route 1 Prediction	Route 2 Prediction
Top 3 features	17.48	19.10	25.48	21.70	18.89	20.85
Top 4 features	17.50	18.95	25.47	21.79	18.89	20.67
Top 5 features	17.50	18.73	25.44	19.02	18.90	20.89
Top 6 features	17.51	18.53	24.78	20.79	18.89	20.86
Top 7 features	17.51	16.89	25.50	20.22	18.89	22.13
Top 8 features	17.51	15.62	25.50	22.01	18.89	19.74
Top 9 features	17.51	15.62	25.50	21.16	18.90	19.75
PEMS data	17.49	19.09	25.54	22.62	18.89	21.70

## Data Availability

Data is contained within the article.
